# Mitochondrial mitophagy in mesenteric artery remodeling in hyperhomocysteinemia

**DOI:** 10.14814/phy2.283

**Published:** 2014-04-23

**Authors:** Anastasia Familtseva, Anuradha Kalani, Pankaj Chaturvedi, Neetu Tyagi, Naira Metreveli, Suresh C. Tyagi

**Affiliations:** 1 Department of Physiology and Biophysics, School of Medicine, University of Louisville, Louisville, 40202, Kentucky

**Keywords:** Endothelial dysfunction, hyperhomocysteinemia, mitochondrial dynamics, oxidative stress

## Abstract

Although high levels of homocysteine also termed as hyperhomocysteinemia (HHcy) has been associated with inflammatory bowel disease and mesenteric artery occlusion, the mitochondrial mechanisms behind endothelial dysfunction that lead to mesenteric artery remodeling are largely unknown. We hypothesize that in HHcy there is increased mitochondrial fission due to altered Mfn‐2/Drp‐1 ratio, which leads to endothelial dysfunction and collagen deposition in the mesenteric artery inducing vascular remodeling. To test this hypothesis, we used four groups of mice: (i) WT (C57BL/6J); (ii) mice with HHcy (CBS+/−); (iii) oxidative stress resistant mice (C3H) and (iv) mice with HHcy and oxidative stress resistance (CBS+/−/C3H). For mitochondrial dynamics, we studied the expression of Mfn‐2 which is a mitochondrial fusion protein and Drp‐1 which is a mitochondrial fission protein by western blots, real‐time PCR and immunohistochemistry. We also examined oxidative stress markers, endothelial cell, and gap junction proteins that play an important role in endothelial dysfunction. Our data showed increase in oxidative stress, mitochondrial fission (Drp‐1), and collagen deposition in CBS+/− compared to WT and C3H mice. We also observed significant down regulation of Mfn‐2 (mitochondrial fusion marker), CD31, eNOS and connexin 40 (gap junction protein) in CBS+/− mice as compared to WT and C3H mice. In conclusion, our data suggested that HHcy increased mitochondrial fission (i.e., decreased Mfn‐2/Drp‐1 ratio, causing mitophagy) that leads to endothelial cell damage and collagen deposition in the mesenteric artery. This is a novel report on the role of mitochondrial dynamics alteration defining mesenteric artery remodeling.

## Abstract (precis)

e00283

This article is a novel report on the role of mitochondrial dynamics in mesenteric artery remodeling during hyperhomocysteinemia. The study can contribute significantly toward understanding the mesenteric mitochondrial mechanisms underpinning inflammatory bowel disease – a major clinical concern.

## Introduction

Clinical studies show that patients with inflammatory bowel disease and mesenteric occlusion are represented with high levels of homocysteine in blood (Gradman et al. 2001; Oussalah et al. 2011). The first report on increased plasma homocysteine levels was by Lamber et al. in patients with Crohn's disease, which is type of inflammatory bowel disease (Lambert et al. 1996). The pathogenesis of these diseases has been attributed to endothelial dysfunction which is due to the production of reactive oxygen species (ROS) (Tyagi et al. 2011) and decrease in nitric oxide (NO) bioavailability (Dranka et al. 2010). Mounting evidence from literature suggests that HHcy is the cause for endothelial dysfunction (Van den Berg et al. 1995; Chambers et al. 1999; Fu et al. 2002), mesenteric artery remodeling and hypertension with robust elevation of metalloproteinase‐ 9 (MMP9) activity, significant collagen deposition, severe loss of elastin, eNOS, and endothelial nitric oxide down regulation (Munjal et al. 2011).

The thiol group of Hcy is auto‐oxidized to reactive oxygen species (ROS), amplifying the level of oxidative stress (OS) (Tyagi et al. 2011) and leading to inflammation. Vathsala et al. have reported that the levels of superoxide and peroxynitrite (OONO^‐^ formed by the interaction of superoxide and nitric oxide) are increased in HHcy in rat aorta and are mediated by NADPH oxidase (NOX) (Edirimanne et al. 2007). The up regulation of NOX activity in vascular cells leads to excessive superoxide ion production under pathological conditions, that lead to increased vessel injury (Mohazzab and Wolin 1994; Mohazzab et al. 1994). The increase in oxidative stress is accompanied by increase in the antioxidant enzyme activities e.g. superoxide dismutase (SOD), which is a mechanism to compensate oxidative stress (Mendes et al. 2010). Hence, the increased levels of superoxide dismutase (SOD) and NOX are indicative of oxidative stress. Apart from oxidative stress, the other factor for endothelial dysfunction is endothelial nitric oxide synthase (the enzyme catalyzing the production of NO from L‐arginine) and decrease in the bioavailability of this enzyme is a hallmark of impaired endothelium. Impaired endothelium with reduced eNOS is incapable to produce vasodilators such as NO for appropriate arteries or arteriole vasodilation in response to an adequate stimulus. Previous studies have reported that HHcy is the cause for endothelial dysfunction (Van den Berg et al. 1995; Chambers et al. 1999; Fu et al. 2002). In addition to eNOS, connexins play important role in maintaining the integrity of endothelium and any disturbance in the levels of connexins indicates disrupted vasomotor tone (De Wit et al. 2000).

Despite abundant literature demonstrating homocysteine‐induced oxidative stress behind mesenteric artery remodeling, the role of mitochondrial dynamics has been explored only a little (Davidson and Duchen 2007; Ganapathy et al. 2011). Mitochondria are the cell organelles whose primary functions are energy production, regulation of cell survival (apoptosis), reactive oxygen species production, and regulation of intracellular Ca^2+^ (Davidson and Duchen 2007). Mitochondria constantly undergo fission and fusion processes in response to physiological stimulus and stress (Pangare and Makino 2012). Mitochondrial fission is the process of mitochondrial disintegration forming two or more separate mitochondrial compartments regulated by dynamin‐related protein 1(Drp‐1), fission‐1(Fis‐1), and mitochondrial fission factor (MFF). Mitochondrial fission process also contributes to quality control by enabling the removal of damaged mitochondria. Elimination of damaged mitochondria is also called mitophagy. On the other hand, mitochondrial fusion is union of two or more mitochondria within a cell to form one, regulated by inner mitochondrial membrane protein optic atrophy‐1(OPA‐1) and two outer mitochondrial membrane proteins: mitofusin‐1and mitofusin‐2 (Mfn‐1, Mfn‐2). Excessive mitochondrial fission and decreased mitochondrial fusion leads to mitochondrial fragmentation. Accumulation of damaged mitochondria and sustained fission status facilitate the release of proapoptic molecules and induce cell apoptosis (Pangare and Makino 2012). Impaired mitochondrial dynamics is also associated with cardiovascular diseases and diabetes (Trudeau et al. 2010; Pangare and Makino 2012; Marzetti et al. 2013; Pradeep and Rajanikant 2013). Although HHcy causes mitochondrial fission (Ganapathy et al. 2011), it remains unclear whether it contributes to endothelial cell damage, nitric oxide, and antioxidant mechanisms. Therefore, we hypothesize that HHcy impairs mitochondrial dynamics increasing mitochondrial fission which leads to endothelial cell damage, endothelial dysfunction, and mesenteric vascular alterations (collagen deposition) (Fig. 8). To test this hypothesis that mitochondrial fusion and fission proteins (Mfn‐2 and Drp‐1) influence mitophagy, along with endothelial damage, and down regulate eNOS and connexins, we used hyperhomocysteinemic (CBS+/−) and oxidative stress‐resistant mice (C3H). This work is novel in terms of defining mitochondrial mitophagic mechanisms underlying mesenteric artery remodeling that is associated with inflammatory bowel disease and hyperhomocysteinemia. Our results pave the way for designing novel therapeutics by regulating mitochondrial dynamics.

## Material and Methods

### Animal models

We used 12‐week‐old mice males of: (i) C57BL/6J (WT); (ii) cystathionine beta synthase deficient mice with HHcy; (iii) C3H/HeJ (C3H), which is resistant to oxidative stress and (iv) CBS+/−/C3H mice, weighting approximately 25–30 g. While the CBS+/− mouse model was used for HHcy, the C3H model was used, as it is resistant to oxidative stress. As per Jackson Laboratories (Bar Harbor, ME) these mice have a mutation in the TLR4 receptor and previous reports show that mice deficient in TLR4 have reduced oxidative stress (Latorre et al. 2013). C3H mice have been used in intestinal inflammation studies (Small et al. 2013). To create CBS+/−/C3H mouse, CBS+/− (male) and C3H (female) were cross‐bred. All animals were fed standard chow and water. The procedures and experiments with mice were reviewed and approved by Institutional Animal Care and Use Committee (IACUC) of the University of Louisville. Four animals per each group were used for this study *n* = 4.

### Genotyping analysis of the WT, CBS+/−, C3H, and CBS+/−/C3H mice

The breeding pair of CBS+/− and C3H/HeJ (C3H) mice was obtained from Jackson Laboratories. After 4 weeks, mice were weaned and genotyped. For genotyping by PCR using specific CBS primers, samples of mice tails were collected. The PCR products were run on 1.2% Agarose gel (prepared in TAE buffer, pH 8.4) with ethidium bromide. The images were recorded in gel documentation system (Bio‐Rad, Hercules, CA) and the genotyping data were confirmed as per Jackson Laboratories Protocol.

### Tissue samples collection

Mesenteric artery tissue samples were obtained from different experimental mice groups washed with 50 mmol/L phosphate buffer saline (PBS) pH of 7.4, frozen in liquid nitrogen and stored at −80°C until use.

### Protein extraction and protein estimation

Mesenteric tissue samples were immersed in ice‐cold RIPA (1 mmol/L) buffer with PMSF and protease inhibitor cocktails (1 *μ*L/mL of lysis buffer, Sigma Aldrich, St. Louis, MO) and got extracted. The extract was centrifuged at 13,400 g for 30 min at 4°C. The supernatant was stored at −80°C till use. Protein estimation was measured by Bradford‐dye (Bio‐Rad, CA) method in 96‐well microtitre plate against Bovine Serum Albumin (BSA) standard. The plate was analyzed at 594 nm in Spectra Max M2 plate reader (Molecular Devices Corporation, Sunnyvale, CA).

### Western blotting

In accordance with protein estimation, mesenteric tissue samples were prepared (60 *μ*g) and loaded onto polyacrylamide gel with SDS in running buffer (Tris‐glycine pH 7.0) in separate wells and run at a constant voltage (110 Volts) until the dye reached to the bottom. Protein transferring was performed by using electro transfer apparatus (Bio‐Rad) with supported nitrocellulose membranes (Bio‐Rad) and blotting buffer for overnight at constant current (120 mA). After blocking for 1 h with 5% nonfat dry milk, the primary antibodies (anti‐, Nox4, SOD‐1, SOD‐2, CBS, eNOS, Mfn‐2 and Drp‐1; Santa Cruz Biotechnology, Dallas, TX; Abcam, Cambridge, MA) were introduced on the membranes for overnight at 4°C. After washing with TBS‐T buffer, membranes were incubated with secondary antibody for 1 h at room temperature (horse radish peroxidase‐conjugated goat anti‐mouse, goat anti‐rabbit, and rabbit anti‐goat IgG with dilution 1:5000 (Santa Cruz, Biotechnology) and antibody for marker. After washing, the antibody–antigen complexes were visualized by using ECL western blotting detection system (GE Healthcare, Piscataway, NJ) and the images were recorded in chemi program of gel documentation system (Bio‐Red, California, USA). The membranes were stripped with stripping buffer (Boston BioProducts, Ashland, MA) blocked with 5% milk for 1 h and incubated with primary antibody for GAPDH (Millipore, Billerica, MA). After washing with TBS‐T, secondary antibody was added (horse radish peroxidase‐conjugated goat anti‐mouse IgG). The GAPDH bands were normalized with different band intensities obtained before, using Bio‐Rad Image Lab densitometry software.

### RT‐PCR

RT‐PCR was used to check the mRNA expression levels of different genes. RNA was isolated with TRIzol^®^ reagent (Invitrogen, Carlsbad, CA) from mesenteric tissues, of different mice groups, according to manufacturer's instructions. Quantification and purity of the RNA was assessed by A260/A280 absorption (one A260 unit equals 50 *μ*g of double‐stranded DNA/mL). Aliquots (2 *μ*g) of total RNA were reverse transcribed into cDNA for 5 min at 70°C using 1 *μ*L oligo (dT15) primers (Invitrogen) in a final volume of 5 *μ*L. To this mixture, 4 *μ*L of 5 mmol/L MgCl2, 4 *μ*L of 5X PCR reaction buffer, 0.5 *μ*L of Im‐Prom‐II^™^ RT, 1 *μ*L 10 mmol/L dNTPs, and 20U of 5 *μ*L rRNasin were added in a total volume of 20 *μ*L. Then the mixture was incubated for 60 min at 42°C, heated to 70°C for 15 min, and cooled to 4°C. Sequence‐specific oligonucleotide primers were prepared commercially (Invitrogen, Mfn2 forward primer 5 ACAGCCTCAGCCGACAGCAT 3′ reverse primer 5' TGCCGAAGGAGCAGACCTT 3′; Drp1 forward primer 5' GCGCTGATCCCGCGTCAT 3′ reverse primer 5′ CCGCACCCACTGTGTTGA 3′;). The PCR was used to analyze different genes (Mfn‐2, Drp‐1, and GAPDH), in a final reaction volume of 25 *μ*L containing 12.5 *μ*L SYBR GREEN PCR master mix, 1 *μ*L cDNA, 40 picomoles of forward, and reverse primers. The mRNA expression was normalized with Rn18s as an internal (housekeeping gene) control along with RT and nontemplate controls. All the experiments were repeated thrice and the data were analyzed statistically (±SD and paired Student's *t*‐test).

### Immunohistochemistry analysis

The mouse was anesthetized and its mesenteric artery was isolated and perfused with 50 mmol/L PBS (pH 7.4). After perfusion, mesenteric artery was filled with tissue freezing medium (Triangle Biomedical Sciences, Durham, NC) and immersed in it to make frozen blocks. The blocks were cut to make 5‐*μ*m tissue sections using cryostat (Leica CM 1850). The slides with tissue sections were blocked for 1 h with permeabilization solution (0.2 g BSA, 50 *μ*L Triton X‐100 in 10 mL PBS (50 mmol/L)). After washing three times in 50 mmol/L PBS for 5 min each, the sections were incubated with primary antibody (anti‐CD31, connexin 40, Mfn‐2, Drp‐1 with the dilution of 1:250) for overnight at 4°C. After that, tissue slides were washed with 50 mmol/L PBS three times for 5 min and incubated with secondary antibody (goat anti‐mouse Alexa flour 488 and goat anti‐rabbit Texas red with dilution 1:500) for 1 h at room temperature. After washing in 50 mmol/L PBS three times for 5 min, the sections were stained with DAPI (1:10,000) for 20 min at room temperature followed by washing in PBS. The tissue sections were mounted with paramount media. The images were obtained by using a laser scanning confocal microscope (×20 objectives, FluoView 1000; Olympus, Center Valley, PA). The images were analyzed by measuring fluorescence intensity with image analysis software (Image Pro‐Plus; Media Cybernetics, Rockville, MD).

### Masson trichrome staining

The same protocol as written above was used to get tissue frozen sections. The slides with tissue were hydrated with distilled water (100, 90 and 70%) and placed in Bouin's Fluid at 56°C for 1 h. After washing with tap water for 5 min, sections were placed in Working Weigert's Iron Hematoxylin Stain (Weigert's Iron Hematoxylin A+ Weigert's Iron Hematoxylin B) for 10 min. After washing in tap water for 10 min, tissue sections were stained in Biebrich Scarlet Acid Fuchsin solution for 7 min. After 30 sec of washing in distilled water, slides were placed in Phosphotungstic Phosphomolybdic Acid solution for 5 min and after that stained in Aniline Blue Stain solution for 7 min. Then tissue sections were placed in 1% Acetic Acid solution for 1 min, rinsed in distilled water for 30 sec, and dehydrated in anhydrous alcohol for 1 min each (70, 90 and 100%). After that, the slides were cleared in clearing reagent (Xyline) three times for 1 min each and mounted. The images were obtained by using light microscopy (×20 objectives, QCapture Pro, Surrey, BC, Canada). The images were analyzed by measuring the color intensity with image analysis software (Image Pro‐Plus; Media Cybernetics).

### Statistical analysis

Quantitative data are expressed as mean ± SEM. Differences between two groups were determined by paired Student's *t*‐test. The results were considered significant if *P* value ≤0.05. For comparing three or more groups, we have used two‐way ANOVA. The *P* value is two‐sided and considered significant if *P* < 0.05 and *F* > *F*_crit._

## Results

### Genotype and phenotype of WT, CBS+/−, C3H, and CBS+/−/C3H mice models

**Figure 1.**
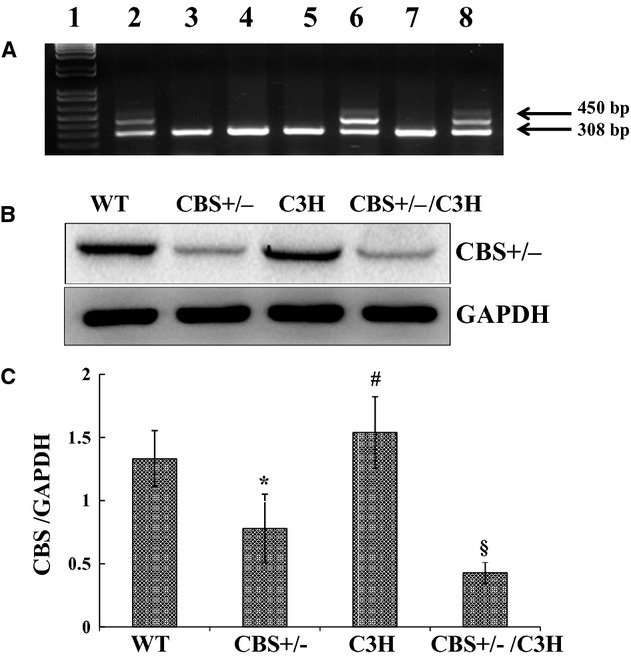
Genotypic and phenotypic analysis of WT, CBS+/− mice, C3H, and CBS+/−/C3H mice: (A) Lane 1: Molecular weight markers; Lane 2, 6&8: CBS+/− bands positioned at 450 and 308 bp; Lane 3, 4, 5&7: CBS+/+, band located at 308 bp; (B) Phenotype of WT, CBS+/−, C3H and CBS+/−/C3H mice with western blot using anti‐ CBS antibody, (C) Bar graphs for CBS protein expression, normalized with GAPDH, *P < 0.05 WT versus CBS+/−, ^#^*P* < 0.05 CBS+/− versus C3H, ^§^*P* < 0.05 WT versus CBS+/−/C3H, *n* = 4.

The genotype analysis was done as per standard protocols from Jackson Laboratory. CBS+/− mice and CBS+/−/C3H mice had two bands located at 450 and 308 bp, while CBS+/+ mice had one band located at 308 bp (Fig. 1A). The results from genotyping were confirmed with Western Blotting via the reduction in cystathionine beta synthase enzyme in CBS+/− and CBS+/−/C3H mice (Fig. 1B and C).

### HHcy evoked oxidative stress in mesenteric artery

**Figure 2.**
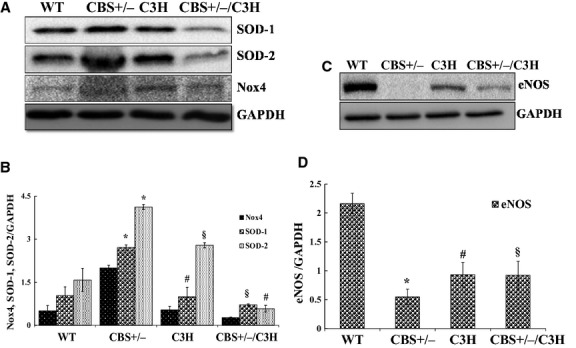
Oxidative stress status in mesenteric artery in HHcy: (A) Western blot analysis of Nox4, SOD‐1 and SOD‐2 protein expressions in WT, CBS+/−, C3H, CBS+/−/C3H mice mesentery. (B) Bar graph for respective protein in mesentery *P < 0.05 WT versus CBS+/−, ^#^*P* < 0.05 CBS+/− versus C3H, ^§^*P* < 0.05 CBS+/− versus CBS+/−/C3H, *n* = 4 per group (for two way ANOVA *F* > *F*_crit._, *F* = 25.71, *P* < 0.001). (C) Western blot analysis of eNOS protein expression in WT, CBS+/−, C3H, and CBS+/−/C3H mice mesentery. (D) Bar plot for eNOS protein expression normalized with GAPDH, *P < 0.05 WT versus CBS+/−, ^#^*P* < 0.05 CBS+/− versus C3H, ^§^*P* < 0.05 CBS+/− versus CBS+/−/C3H.

We found that Nox4 (oxidative stress marker), SOD‐1, and SOD‐2 (antioxidants) were up regulated in mesentery of CBS+/− mice compared to WT mice. The protein expressions of Nox4 and SOD‐1 were down regulated in C3H as in CBS+/−/C3H compared to CBS+/− mice. Interestingly, the expression of SOD‐2 (mitochondrial antioxidant) was increased in C3H compared to CBS+/−/C3H mice (Fig. 2A and B). We also noticed that eNOS was significantly down regulated in CBS+/− compared to WT mice (Fig. 2C and D). Moreover, the protein expression of eNOS was up regulated in mesentery of CBS+/−/C3H and C3H compared to CBS+/− mice.

### Altered mitochondrial dynamics in HHcy

**Figure 3.**
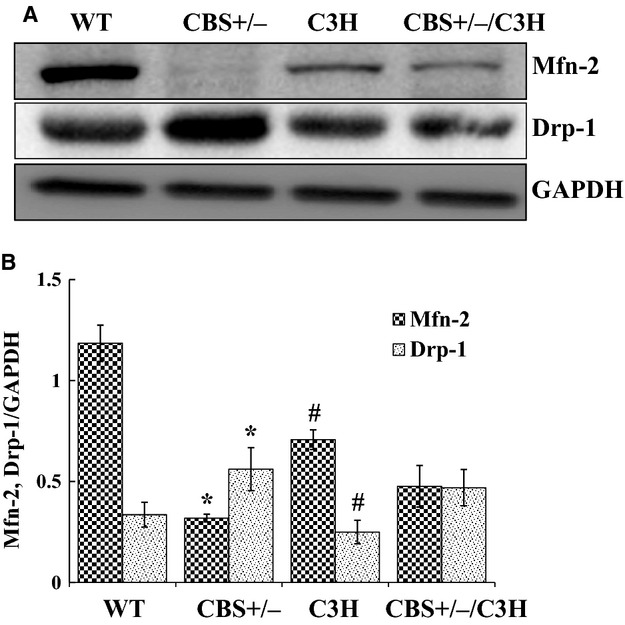
Mitochondrial dynamics in mesenteric artery in HHcy: (A) Western blot analysis of Mfn‐2 and Drp‐1 protein expressions in WT, CBS+/−, C3H, and CBS+/−/C3H mice mesentery. (B) Bar graph for Mfn‐2 and Drp‐1 protein expressions in mesentery, normalized with GAPDH, *P < 0.05 WT versus CBS+/−, ^#^*P* < 0.05 CBS+/− versus C3H (for two way ANOVA *F* > *F*_crit._, *F* = 21.62, *P* < 0.0019).

**Figure 4.**
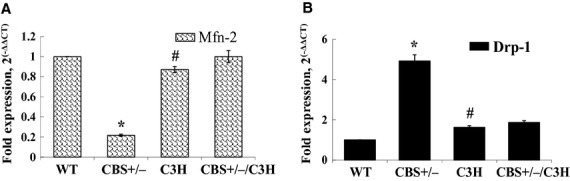
Alteration of mitochondrial dynamics in mesenteric artery in HHcy: (A) Real‐time expression of Mfn‐2 mRNA in mesentery *P < 0.05 WT versus CBS+/−, ^#^*P* < 0.05 CBS+/− versus C3H; (B) Real‐time expression of Drp‐1 mRNA in mesentery *P < 0.05 WT versus CBS+/−, ^#^*P* < 0.05 CBS+/− versus C3H.

**Figure 5.**
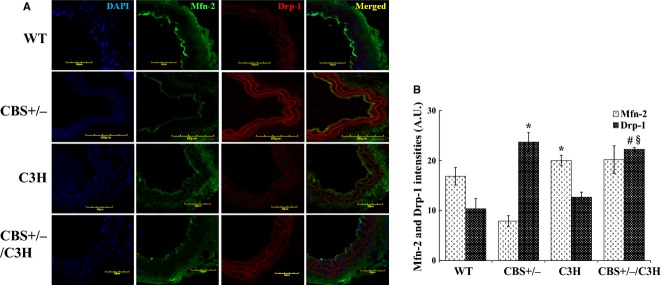
Increased mitochondrial fission in HHcy: (A) Mfn‐2 and Drp‐1 intensities in WT, CBS+/−, C3H, and CBS+/−/C3H mesenteric arteries with ×20 magnification. (B) Bar graph for Drp‐1 expression in mesenteric artery. Data expressed in arbitrary units. *P < 0.05 WT versus CBS+/−, ^#^*P* < 0.05 WT versus CBS+/−C3H, ^§^*P* < 0.05 C3H versus CBS+/−/C3H; for Mfn‐2 expression *P < 0.05 CBS+/− versus C3H.

To evaluate the effect of HHcy and oxidative stress on mitochondrial dynamics, we analyzed two major proteins: Mfn‐2 (regulates mitochondrial fusion) and Drp‐1 (regulates mitochondrial fission) by western blot, PCR and immunohistochemistry. Western blot data (Fig. 3A and B) showed that the protein expression of Mfn‐2 was decreased in CBS+/− compared to WT mice. In addition, Mfn‐2 was increased in C3H as compared to CBS+/−/C3H mice. Drp‐1 protein expression was significantly up regulated in CBS+/− mice as compared to WT mice. The fission marker was also increased in CBS+/−/C3H mice as compared to C3H mice. Using real‐time PCR (Fig. 4A and B), we confirmed that Mfn‐2 was also down regulated in CBS+/− mice as compared to WT and CBS+/−/C3H mice and Drp‐1 was also up regulated in CBS+/− mice as compared to WT and CBS+/−/C3H mice. By using immunohistochemistry (Fig. 5A and B), we determined that the intensity of Mfn‐2 was decreased in mesenteric artery of CBS+/− mice as compared to WT, CBS+/−/C3H mice. In addition, the intensity of Drp‐1 in mesenteric artery was increased in CBS+/− and CBS+/−/C3H mice as compared to WT mice. These results suggested the prevalence of mitochondrial fission over mitochondrial fusion due to HHcy in CBS+/− mice which is the main cause of mitophagy.

### HHcy‐induced endothelial cell layer damage

To determine whether elevated homocysteine level impaired endothelial cell layer, CD31 and connexin 40 intensity levels were observed (6A, 6B). By using immunohistochemistry, we determined that the intensity of CD31 was decreased in CBS+/− mice as compared to WT, C3H, and CBS+/−/C3H mice. The intensity of connexin 40 (gap junction's protein in endothelial cells) was significantly reduced in CBS+/− and CBS+/−/C3H mice as compared to WT and C3H mice.

### HHcy caused collagen deposition in mesenteric artery

To observe collagen content, Masson Trichrome staining was performed (Fig. 7A and B). The collagen was significantly up regulated in CBS+/− mice as compared to WT, C3H, and CBS+/−/C3H mice. Moreover, we found that collagen expression was reduced in CBS+/−/C3H mice as compared to CBS+/− mice.

## Discussion

In the present study, we observed the increase in the expression of Nox4 (oxidative stress marker), SOD‐1 (antioxidant marker), and SOD‐2 (mitochondrial antioxidant marker) in CBS+/− deficient mice as compared to WT, C3H, and CBS+/−/C3H mice (Fig. 2A and B). The oxidative stress is the main player in the hyperhomocysteinemia pathogenesis. Many previous studies indicated the role of oxidative stress in HHcy and correlation of these two pathological conditions with cardiovascular, cerebrovascular, and renovascular diseases (Jung et al. 1832; Tyagi et al. 2005; Mishra et al. 2010; Kamat et al. 2013). With these findings, we assume that during acute phase of HHcy following oxidative stress the defensive mechanisms will be activated to abate oxidative damage and balance the biological system. However, during chronic stage of the pathological condition, the defense will be exhausted and would cause antioxidant down regulation, which may explain the early findings (Givvimani et al. 2012a). In the meantime, previous study also found increase in SOD‐1 and SOD‐2 during oxidative stress (Park et al. 2013) or HHcy (Wilcken et al. 2000; Mendes et al. 2010). Interestingly, SOD‐2 was also up regulated in C3H mice, which may suggest that this mouse strain has antioxidant nature and potentially more resistant to oxidative stress compare to different strains, however, the mechanism is unknown.

**Figure 6.**
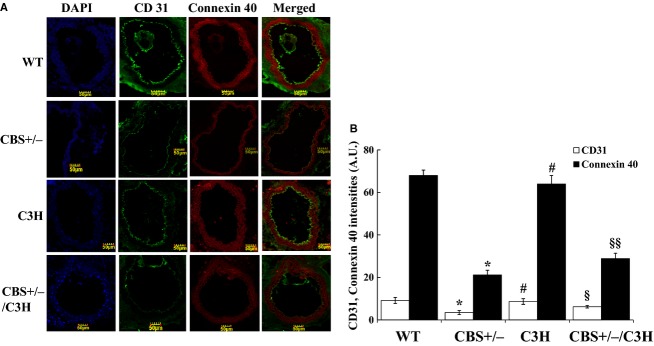
Immunohistochemistry of mesenteric artery in different mouse groups: (A) CD31 and Connexin 40 intensities in WT, CBS+/−, C3H and CBS+/−/C3H mesenteric arteries, ×20 magnification. (B) Bar graph for respective proteins in mesenteric artery. Data expressed in arbitrary units. *P < 0.05 WT versus CBS+/−, ^#^*P* < 0.05 CBS+/− versus C3H, ^§^*P* < 0.05 CBS+/− versus CBS+/−/C3H, ^§§^*P* < 0.05 WT versus CBS+/−/C3H, and C3H versus CBS+/−/C3H.

**Figure 7.**
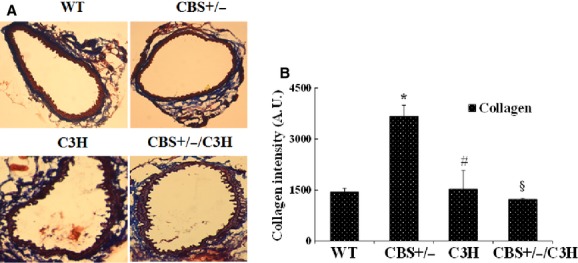
Masson Trichrome staining of mesenteric artery in different mouse strains: (A) Collagen intensity in mesenteric artery in WT, CBS+/−, C3H, and CBS+/−/C3H mice, ×20 magnification. (B) Bar graph for collagen expression in mesenteric arteries. Data expressed in arbitrary units. *P < 0.05 WT versus CBS+/−, ^#^*P* < 0.05 CBS+/− versus C3H, ^§^*P* < 0.05 CBS+/− versus CBS+/−/C3H.

Mounting evidences suggest that HHcy and oxidative stress impair endothelial cell layer which leads to endothelial nitric oxide synthase down regulation and significant reduction in endothelium‐dependent vasorelaxation (Chen et al. 2002; Fu et al. 2002; Qipshidze et al. 2011). Our findings showed a significant eNOS down regulation in CBS+/− mice as compared to WT, C3H, and CBS+/−/C3H mice (Fig. 2C and D). In addition, we observed a significant decrease in CD31 (endothelial cell marker) and connexin 40 in CBS+/− mice as compared to WT, C3H, and CBS+/−/C3H mice (Fig. 6A and B). Connexin 40 is a gap junction protein which is highly expressed in the endothelial cells. Connexin 40 deficient rodents developed arterial hypertension and have impaired intercellular signaling with altered propagation of vasodilation (De Wit et al. 2000; Heil et al. 2004; Mather et al. 2005). On the contrary, Munjal et al. reported connexin 40 up regulation in endothelial cells during HHcy and suggested that HHcy leads to mesenteric arterial remodeling by activation of MMP‐9 and collagen deposition (Munjal et al. 2011). In agreement to this study, we showed significant deposition of collagen in CBS+/− mice (which are hyperhomocysteinemic), as compared to WT, C3H, and CBS+/−/C3H mice (Fig. 7A and B).

**Figure 8.**
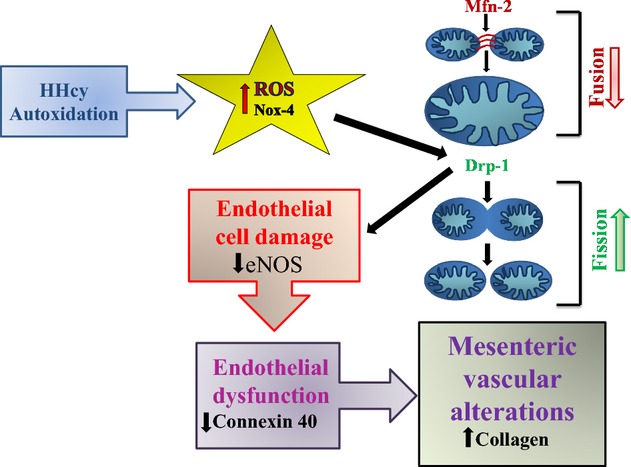
Schematic representation of hypothesis: Hyperhomocysteinemia and oxidative stress alter mitochondrial dynamics by decreasing mitochondrial fusion and increasing fission, which leads to endothelial cell damage, mesenteric artery dysfunction, and vascular alterations with collagen accumulation.

In the present study, we aimed to understand the effect of HHcy on mitochondrial dynamics in mesenteric artery (Fig. 3A and B; Fig. 4A and B; Fig. 5A and B). It has been reported that mitochondria constantly undergo fusion (regulated by OPA‐1, Mfn‐1, and Mfn‐2) and fission (regulated by Drp‐1, Fis‐1,and MFF) in response to physiological stimuli or stress. In addition, excessive mitochondrial fission and decreased mitochondrial fusion leads to mitochondrial fragmentation and accumulation of damaged mitochondria which initiates mitophagy or cell apoptosis (Dranka et al. 2010; Makino et al. 2010; Shenouda et al. 2011; Pangare and Makino 2012). Hyperhomocysteinemia promotes intense mitochondrial fission and decreases mitochondrial fusion facilitating cell death (Ganapathy et al. 2011). We found a significant down regulation of Mfn‐2 (mitochondrial fusion marker) in CBS+/− deficient mice as compared to WT and C3H mice. In addition, Drp‐1 (mitochondrial fission marker) was significantly up regulated in CBS+/− deficient mice as compared to WT and C3H mice. Interestingly, Mfn‐2 was increased in CBS+/−/C3H mice as compared to CBS+/− mice. In addition, Drp‐1 protein was decreased in CBS+/−/C3H mice as compared to CBS+/− mice.

It has been showed by our group that Drp‐1 inhibitor (Mdivi‐1) prevented mitochondrial fission, apoptosis, loss of mitochondrial membrane potential (MMP), and cell death and promoted angiogenesis (Givvimani et al. 2012b). Other studies emphasized the role of Drp‐1 inhibitors (Drp1 siRNA) in prevention of mitochondrial fission, loss of mitochondrial membrane potential, and cell death (Grohm et al. 2012). Overexpression of DRP‐1 leads to mitochondrial membrane potential loss, cytochrome c release, caspase‐3 activation, and generation of reactive oxygen species (ROS) causing cell apoptosis. In contrast, dominant‐negative suppression of DRP‐1 function significantly prevents cell apoptosis (Peng et al. 2012). Hence, in this study, we focused on the role of Mfn2 and Drp1 proteins in mesenteric artery the imbalance of which leads to mitophagy, inducing cell damage following mesenteric artery remodeling (Fig. 8). Our findings indicated increased mitochondrial fission as a novel mechanism of homocysteine toxicity to mesenteric artery.

## Conclusions

These findings indicate the dominance of endothelial cell mitochondrial fission over mitochondrial fusion in HHcy and oxidative stress; which may explain the endothelial cell loss and dysfunction that follows collagen deposition in mesenteric artery.

## Conflict of Interest

None declared.
